# Evaluation of Two Influenza Surveillance Systems in South Africa

**DOI:** 10.1371/journal.pone.0120226

**Published:** 2015-03-30

**Authors:** Eric Budgell, Adam L. Cohen, Jo McAnerney, Sibongile Walaza, Shabir A. Madhi, Lucille Blumberg, Halima Dawood, Kathleen Kahn, Stefano Tempia, Marietjie Venter, Cheryl Cohen

**Affiliations:** 1 Nuffield Department of Population Health, University of Oxford, Oxford, United Kingdom; 2 Influenza Division, Centers for Disease Control and Prevention, Atlanta, United States and Influenza Program, Centers for Disease Control and Prevention—South Africa, Pretoria, South Africa; 3 Centre for Respiratory Diseases and Meningitis, National Institute for Communicable Diseases of the National Health Laboratory Services, Johannesburg, South Africa; 4 Department of Science and Technology/National Research Foundation: Vaccine-Preventable Diseases, University of the Witwatersrand, Johannesburg, South Africa; 5 Department of Medicine, Pietermaritzburg Metropolitan Hospital Complex and University of KwaZulu Natal, South Africa; 6 MRC/Wits Rural Public Health and Health Transitions Research Unit (Agincourt), School of Public Health, Faculty of Health Sciences, University of the Witwatersrand, Johannesburg, South Africa; 7 CTS Global, Inc., Los Angeles, United States of America; 8 Zoonoses Research Unit, Department of Medical Virology, University of Pretoria, Pretoria, South Africa; 9 Schools of Public Health and Pathology, University of the Witwatersrand, Johannesburg, South Africa; University Hospital San Giovanni Battista di Torino, ITALY

## Abstract

**Background:**

The World Health Organisation recommends outpatient influenza-like illness (ILI) and inpatient severe acute respiratory illness (SARI) surveillance. We evaluated two influenza surveillance systems in South Africa: one for ILI and another for SARI.

**Methodology:**

The Viral Watch (VW) programme has collected virological influenza surveillance data voluntarily from patients with ILI since 1984 in private and public clinics in all 9 South African provinces. The SARI surveillance programme has collected epidemiological and virological influenza surveillance data since 2009 in public hospitals in 4 provinces by dedicated personnel. We compared nine surveillance system attributes from 2009–2012.

**Results:**

We analysed data from 18,293 SARI patients and 9,104 ILI patients. The annual proportion of samples testing positive for influenza was higher for VW (mean 41%) than SARI (mean 8%) and generally exceeded the seasonal threshold from May to September (VW: weeks 21–40; SARI: weeks 23–39). Data quality was a major strength of SARI (most data completion measures >90%; adherence to definitions: 88–89%) and a relative weakness of the VW programme (62% of forms complete, with limited epidemiologic data collected; adherence to definitions: 65–82%). Timeliness was a relative strength of both systems (e.g. both collected >93% of all respiratory specimens within 7 days of symptom onset). ILI surveillance was more nationally representative, financially sustainable and expandable than the SARI system. Though the SARI programme is not nationally representative, the high quality and detail of SARI data collection sheds light on the local burden and epidemiology of severe influenza-associated disease.

**Conclusions:**

To best monitor influenza in South Africa, we propose that both ILI and SARI should be under surveillance. Improving ILI surveillance will require better quality and more systematic data collection, and SARI surveillance should be expanded to be more nationally representative, even if this requires scaling back on information gathered.

## Introduction

Annual influenza epidemics are estimated to cause 3–5 million cases of severe disease and 250,000–500,000 deaths globally, with the highest risk of severe disease occurring in adults older than 65 years, children younger than 2 years, pregnant women, and persons with certain medical conditions[1–2]. Seasonal influenza is an important viral cause of pneumonia in children[3], and mortality rates due to pneumonia in children are highest in Africa[4].

Despite recent progress in describing the epidemiology and burden of influenza in sub-Saharan Africa[5], most countries in this region lack longitudinal national surveillance data needed to inform prevention and control strategies[6]. With little robust influenza data available, the impact of the disease in this region remains poorly understood.

In South Africa, where influenza circulates seasonally during the Southern Hemisphere winter[7], rates of seasonal influenza-related excess mortality in adults ≥65 years have been estimated to be at least three times higher than in the United States[8]. Widespread co-morbidities such as human immunodeficiency virus (HIV) have also been identified as increasing the risk of severe influenza-associated disease[9–10]. An interim report of influenza A(H1N1)pdm09 deaths in South Africa revealed that more than half occurred among individuals with HIV and 10% occurred in individuals with active tuberculosis[11].

Historically, influenza surveillance has been conducted through sentinel surveillance for influenza-like illness (ILI), with respiratory specimens being collected for virological monitoring and vaccine strain selection. The 2009 influenza pandemic highlighted the need for improved surveillance for severe influenza-associated disease and the use of standardized approaches to data collection and reporting. In response to this need, several international organizations have partnered with African governments to invest in the development of epidemiological and laboratory influenza surveillance capacity.

The World Health Organization (WHO) now recommends countries to perform surveillance for both ILI and influenza-associated severe acute respiratory infection (SARI), and that surveillance systems undergo a comprehensive evaluation periodically, beginning 1–2 years after implementation and before adding new sentinel sites[12]. To ensure national surveillance objectives are being met, we conducted an evaluation and comparison of two influenza surveillance systems in South Africa: a long-standing ILI system and a newer SARI system.

## Methods

### Surveillance systems

We described the surveillance characteristics of the Viral Watch Programme and SARI surveillance programmes (Table 1).

**Table 1:** Characteristics of the Viral Watch and severe acute respiratory infection (SARI) surveillance programmes.

Surveillance characteristics	Viral Watch	SARI Programme
**Syndrome**	Influenza-like Illness (ILI)	Severe acute respiratory infection (SARI)
**Data collection**	Active, prospective, performed on a voluntary basis by participating sentinel sites	Active, prospective, performed by dedicated personnel
**In-patient vs. Out-patient**	Out-patient, ambulatory clinics	In-patient, hospital-based
**Public vs. Private**	Public and private*	Public
**Geographic scope**	All 9 provinces	4 out of 9 provinces
**Number of sentinel sites**	205	6
**Coordinating body**	National Institute for Communicable Diseases (NICD)	National Institute for Communicable Diseases (NICD), supported by CDC
**Source of funding**	NICD	NICD & CDC
	Primarily virologic surveillance objectives:	Both epidemiologic and virologic surveillance objectives:
	Determine the relative contribution of influenza and other respiratory pathogens to ILI	Determine the relative contribution of influenza and other respiratory pathogens to SARI
	Monitor the type and subtype of circulating influenza viruses and other respiratory viruses	Monitor the type and subtype of circulating influenza viruses and other respiratory viruses
	Provide baseline data on the seasonality and distribution of influenza and other respiratory viruses	Provide baseline data on the seasonality and distribution of influenza and other respiratory viruses
**Surveillance objectives**	Inform strain selection for the southern hemisphere influenza vaccine	Describe trends in SARI incidence and case-fatality rates
	Act as a platform for studying the effectiveness of influenza vaccines	Describe the epidemiological characteristics of SARI cases and identify high risk groups
	Monitor antiviral sensitivity to inform the clinical use of antiviral therapies	Describe the burden of SARI across age and risk groups
	Detect novel respiratory viruses	Estimate the severity of influenza epidemics
		Monitor antiviral sensitivity to inform the clinical use of antiviral therapies
		Detect novel respiratory viruses
		Act as a platform for studying the effectiveness of vaccines

* Most sentinel sites consist of private sector general practitioners, though primary care clinics, paediatric outpatient departments, and occupational health clinics are also included.

#### Viral Watch Programme

**Fig 1:**
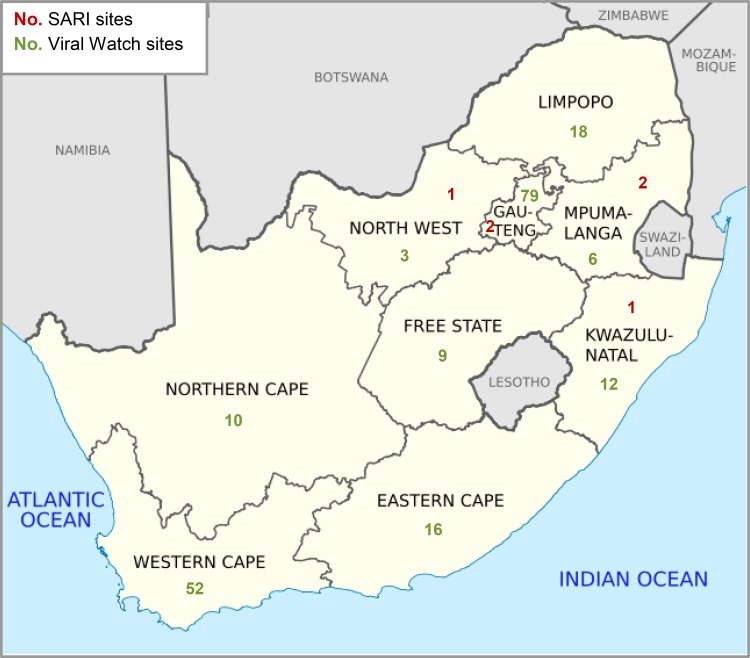
Map showing numbers of Viral Watch and SARI sentinel sites, by province 2012

Established in 1984, the Viral Watch (VW) programme is an active, prospective surveillance programme run by volunteer medical practitioners to monitor outpatient influenza. ILI surveillance is performed at 205 sentinel sites in private and public clinics in all 9 of South Africa’s provinces (Fig. 1)[7]. A case of ILI is defined as: an acute respiratory infection with a measured fever (≥38°C), AND cough, AND onset within the past 7 days. Sites are requested to enrol patients with onset of symptoms within 72 hours of presentation, and to enrol no more than 5 patients per week. Nose and throat swabs are collected from consenting patients and most samples (>80%) are sent directly to the National Institute for Communicable Diseases (NICD) for influenza testing by real-time reverse transcription polymerase chain reaction (rRT-PCR). Specimens from selected provinces are first tested for influenza by shell viral culture at local laboratories, after which positive samples are shipped to NICD for influenza subtyping by rRT-PCR. NICD is a current WHO National Influenza Centre[13] and VW is fully funded by NICD.

#### SARI Surveillance

SARI surveillance is performed by teams of dedicated personnel at 6 sentinel sites in public hospitals in 4 provinces (Fig. 1)[9]. Established in February 2009, this active, prospective, hospital-based surveillance programme collects detailed epidemiological and virological influenza surveillance data. In persons ≥5 years of age, a case of SARI is defined as: an acute respiratory infection with a history of fever or measured fever (≥38°C), AND cough, AND onset within the past 7 days, AND requiring hospitalization. In children 3–59 months of age, a case of SARI is defined as physician-diagnosed lower respiratory tract infection (LRTI) with onset in the past 7 days and requiring hospitalization, while for infants aged 2 days to <3 months the definition includes suspected sepsis or LRTI irrespective of signs and symptoms with onset in the past 7 days. All patients admitted on week days are eligible for enrolment, except adult patients at Chris Hani Baragwanath Hospital, where systematic sampling is used on 2 of every 5 week days (selected days rotate) due to high patient load and resource constraints. Specimens collected include nasopharyngeal aspirates from patients <5 years of age and nasopharyngeal and throat swabs from patients ≥5 years of age. Respiratory specimens are transported on ice to NICD within 72 hours of collection and tested for 10 respiratory viruses, including influenza A and B viruses, by multiplex rRT-PCR[14]. Influenza virus subtypes are also identified by rRT-PCR. The SARI programme is coordinated by NICD with financial and technical assistance provided by the U.S. Centers for Disease Control and Prevention (CDC).

### Ethics Statement

The SARI surveillance protocol was approved by the Research Ethics Committees of the Universities of the Witwatersrand and KwaZulu-Natal. Ethical approval for the Viral Watch project was obtained from the University of the Witwatersrand Research Ethics Committee. This surveillance was deemed non-research by the U.S. CDC. Patient information was anonymized and de-identified prior to analysis.

### Evaluation and comparison of surveillance systems

#### I. Influenza seasonality and circulating strains

For each system we describe the weekly proportion of samples testing positive for influenza, the week of peak detection, and season start and end dates (seasonal threshold defined as influenza detection rates ≥10% and <10% for two consecutive weeks, respectively) from 2009 to 2012. The number of respiratory specimens tested by week and the proportion testing positive for influenza and influenza types and subtypes was also assessed. Only samples tested by rRT-PCR were included in our analysis.

#### II. Surveillance system attributes

CDC guidelines[15] suggest the usefulness of a surveillance system is dependent on the actions that can be taken as a result of data collection and analysis; specifically, whether the system is able to: (1) guide disease prevention and control activities through the timely detection of adverse health-events, (2) estimate the magnitude of morbidity and mortality and associated risk factors, (3) detect trends that signal changes in incidence, including epidemics, (4) permit assessment of prevention and control measures, (5) lead to improved health and social policy or clinical practice, and (6) stimulate research to inform prevention and control measures. Using criteria established by the CDC[15], we assessed nine surveillance system attributes that can affect usefulness, including quantitative analyses of data quality, timeliness, sensitivity, and positive predictive value, and qualitative descriptive analyses of representativeness, simplicity, flexibility, acceptability, and stability.

Data quality was assessed by measuring the completeness of patient interview forms, form transmission, respiratory specimen collection and testing, and by checking patient sign and symptom records and primary diagnosis to determine whether surveillance case definitions had been adhered to properly. Data collected by each system was also compared against minimum data collection standards for ILI and SARI surveillance (S1 Table)[12].

Timeliness was assessed by measuring the duration of time for the collection, transfer, and processing of forms and specimens, and for the availability of laboratory results.

Sensitivity, specificity, positive predictive value (PPV), and negative predictive value (NPV) were obtained for one SARI and two ILI case definitions for predicting influenza infection (Table 2). The clinical case definitions selected for this comparison included those used by each system for screening and enrolment, and an older ILI case definition used by VW prior to March 2012.

**Table 2:** Sensitivity, specificity, positive predictive value (PPV), and negative predictive value (NPV) of selected SARI and ILI sign and symptom combinations for laboratory-confirmed influenza infection, South Africa, February 2009–April 2012.

Case Definition	Comparison group	Number tested	Sensitivity % (95% CI)	Specificity % (95% CI)	PPV % (95% CI)	NPV % (95% CI)
SARI case definitions*						
Any child aged 2 days to < 3 months with (i) suspected sepsis OR physician diagnosed LRTI irrespective of signs and symptoms, AND (ii) onset within the past 7 days (in use from February 2009 to present)	Did not meet case definition	812	89.6 (82.4, 94.1)	10.8 (8.7, 13.3)	13.1 (10.8, 15.8)	87.4 (78.8, 92.8)
Any child aged 3–59 months with (i) LRTI, including bronchiolitis or pneumonia or bronchitis or pleural effusion, AND (ii) onset within the past 7 days (in use from February 2009 to present)	Did not meet case definition	2,486	94.3 (91.4, 96.2)	12.9 (11.5, 14.4)	15.8 (14.4, 17.4)	92.9 (89.3, 95.3)
ILI case definitions*						
Patients of all age groups, with (i) acute respiratory tract infection of recent onset (within 72 hours), AND (ii) sudden onset of fever, AND (iii) two or more of headache, myalgia, cough, sore throat (in use from 2009 to February 2012)	Did not meet case definition	4,914	88.7 (87.3, 90.0)	22.2 (20.7, 23.8)	45.9 (44.4, 47.4)	72.5 (69.4, 75.4)
Patients of all age groups, with an acute respiratory illness with (i) measured fever of ≥38°C, AND (ii) cough, AND (iii) onset within the past 7 days (in use from March 2012 to present)	Did not meet case definition	4,914	89.7 (88.3, 90.9)	28.7 (27.0, 30.4)	48.3 (46.8, 49.9)	78.8 (76.2, 81.2)

*Only respiratory specimens collected during influenza seasons (May to September) were included in this analysis.

## Results

### I. Influenza seasonality and circulating strains

**Fig 2:**
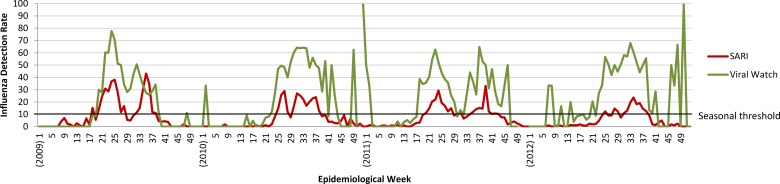
Laboratory confirmed influenza detection rates, South Africa (2009–2012)*

During 1 January 2009 to 31 December 2012, we analysed samples from 18,293 SARI patients and 9,104 ILI patients. On average, the proportion of samples testing positive for influenza exceeded the seasonal threshold from May to September (VW: weeks 21–40 with mean peak in week 30; SARI: weeks 23–39 with mean peak in week 31) and was consistently higher for VW (mean 41%, mean annual range: 38–43%) than SARI (mean 8%, mean annual range: 5–11%) in all four years analysed [relative risk (RR): 5.2, 95% CI: 5.0–5.5, p<.0001; Fig. 2]. Although neither system is used to estimate an epidemic threshold, both are considering doing so in the future.

*Weeks with small numbers of samples are prone to widely varying influenza detection rates.

The circulating influenza strains detected were similar in proportion between VW and SARI systems, except in 2011 when VW identified more A(H1N1)pdm09 (75%) and less influenza B (11%) than SARI (A(H1N1)pdm09: 39%; influenza B: 36%) (S2 Table).

**Fig 3:**
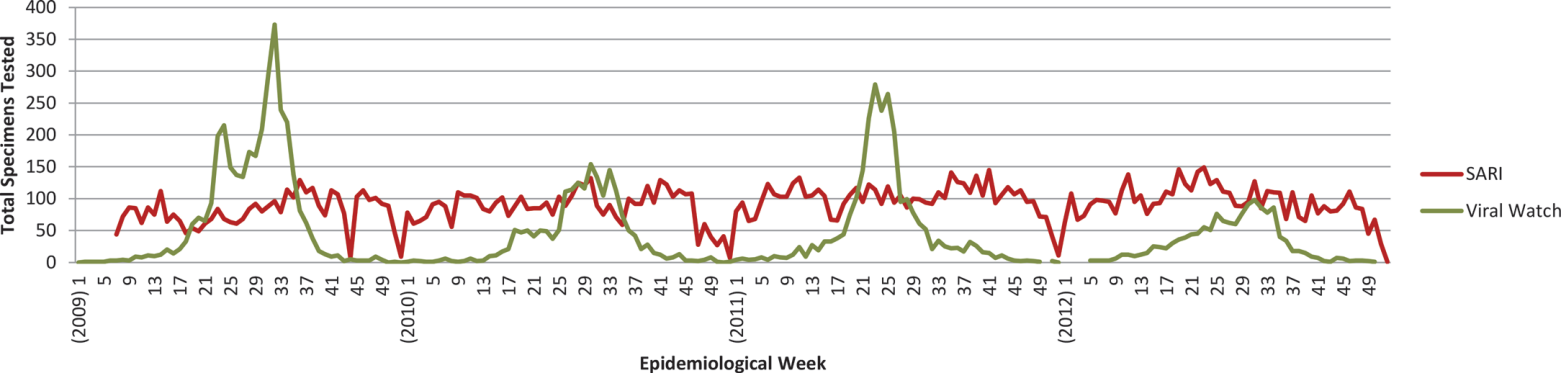
Number of respiratory specimens tested by week, South Africa (2009–2012).

VW showed greater variability in the number of specimens tested per week (median 15, interquartile range (IQR): 3–54) than the SARI system (median 94, IQR: 75–108), and unlike SARI, VW specimen collection declined rapidly outside the annual influenza season (Fig. 3). VW also tested fewer specimens per year (mean 2,276, range: 1,398–3,351) than SARI (mean 4,709, range: 3,658–5,202), though this may be partly due to the weekly enrolment limits placed on VW sentinel sites.

### II. Surveillance system attributes

The nine surveillance system attributes were analysed prior to the end of the 2012 influenza season, using surveillance data collected between 01 January 2009 and 30 June 2012 from 15,271 SARI patients and 8,654 ILI patients.

The relative strengths and weaknesses of the SARI and VW systems are shown in Table 3.

**Table 3:** Strengths and limitations of surveillance attributes, SARI and Viral Watch surveillance programmes, South Africa.

System Attribute	Definition	Viral Watch surveillance	SARI surveillance
**Representativeness**	Extent to which health-related events are accurately described over time and within populations	Relative Strength	Relative Weakness
**Simplicity**	System design and ease of operation	Major Strength	Relative Weakness
**Flexibility**	Adaptability to changing information needs or operating conditions	Major strength	Relative Strength
**Acceptability**	Willingness of persons and organizations to participate in system	Relative Strength	Relative strength
**Stability**	Reliability and availability of the system	Relative Strength	Relative Strength
**Data Quality**	Data completeness and validity	Relative Weakness	Major Strength
**Timeliness**	Time interval between data reporting steps	Relative Strength	Relative Strength
**Sensitivity**	The proportion of true cases detected by case definitions and diagnostic tests	Relative Strength	Relative Strength
**Positive Predictive Value (PPV)**	Proportion of reported cases that are true cases	Relative Strength	Relative Weakness

#### Data Quality

The SARI system collects detailed epidemiological patient data; when compared against minimum data collection standards for ILI and SARI surveillance (S1 Table), we found patient interview forms contained all data elements recommended by WHO[12]. By contrast, VW collects minimal epidemiological patient data. When compared against minimum data collection standards for ILI surveillance, we found VW does not collect data on the use of antivirals for current illness, or pre-existing conditions such as chronic neurological or neuromuscular disease and haematological disorders.

Data quality was a major strength of the SARI system, with 40/43 key data elements on patient interview forms having completeness measures above 90% (n = 15,189 forms). Completeness of other SARI forms (i.e., lab slips, Hospital Results Forms, Final Outcome Forms) were similarly high, as were measures of form transmission (98% of enrolled patients had all surveillance forms transferred from sentinel sites to NICD), data capturing (>99.9% of all forms received at NICD were captured by data entry clerks), and specimen collection and testing (98% of SARI patients had respiratory specimens collected and tested for influenza). Using available data on primary diagnosis, adherence to the SARI case definition (S3 Table) in persons aged 2 days to <3 months was at least 89% (725/812) and adherence in persons aged 3–59 months was at least 88% (2,192/2,486). We were not able to evaluate adherence to the SARI case definition in persons aged 5 years or older because the appropriate data were not routinely collected during the study timeframe.

Data quality was a relative weakness of VW. Over the four years analysed, 62% (n = 8,556) of patient interview forms had complete data available for all data elements, though completeness improved with new forms introduced in March 2012 (83% between April–June 2012, n = 698). Measures of specimen collection and testing were very high; 100% of ILI patients had respiratory specimens collected and tested for influenza, and 99% (n = 2,667) of influenza A results were subtyped. Adherence to the ILI case definition in use from 2009 to March 2012 was 82% (n = 5,447), but decreased to 65% (n = 592) with the introduction of a new ILI case definition in March 2012.

#### Timeliness

Timeliness was a relative strength of the SARI programme; 93% (12,835/13,813) of patients had respiratory specimens collected within 7 days of symptom onset (median: 3 days, IQR: 2–5 days), and 95% (13,291/14,020) of patients had respiratory specimens collected within 1 day of hospital admission. The time interval from specimen collection to capturing of results into analytic datasets at NICD was a median of 8 days (IQR: 7–11, n = 7,559). There were weaknesses, however; only 51% (7,770/15,243) of case investigation forms were received at NICD within the programme target of 14 days after patient interviews (median: 14 days, IQR: 7–28).

Timeliness was also a relative strength of VW; 98% (n = 5,659) of ILI cases had respiratory specimens collected within 7 days of symptom onset (median: 1 day), and the median time from specimen collection to receipt at NICD is 1 day (n = 7,744). Once at NICD, however, the median time to availability of laboratory results was 4 days (n = 612), which exceeded the programme target by 1 day. Although case investigation forms for both systems are currently paper-based, electronic data collection is being considered and may help to improve the timeliness of future data transfer and capturing.

#### Sensitivity and PPV

The SARI programme’s SARI case definition in persons aged 2 days to <3 months was introduced in February 2009 (S3 Table); using incorrectly enrolled patients who did not meet the case definition as a comparator, we found the sensitivity of identifying laboratory-proven influenza was high (89.6%; 95% CI: 82.4–94.1) and specificity (10.8%; 95% CI: 8.7–13.3) and PPV were low (13.1%; 95% CI: 10.8–15.8). Similarly, in children aged 3–59 months, using enrolled patients who did not meet the case definition as a comparator, we found sensitivity was high (94.3%; 95% CI: 91.4–96.2) and specificity (12.9%; 95% CI: 11.5–14.4) and PPV were low (15.8%; 95% CI: 14.4–17.4). We were not able to evaluate sensitivity and PPV of SARI case definitions in persons aged 5 years or older because the appropriate data was not routinely collected during the study timeframe.

The VW’s current ILI case definition was introduced in March 2012 (S3 Table). Using incorrectly enrolled patients who did not meet the case definition as a comparator, this slightly improved sensitivity (89.7%; 95% CI: 88.3–90.9) and specificity (28.7%; 95% CI: 27.0–30.4) of identifying laboratory-proven influenza compared with the ILI case definition in use prior to March 2012 [sensitivity 88.7% (95% CI: 87.3–90.0), specificity 22.2% (95% CI: 20.7–23.8)] (Table 2). PPV estimates were somewhat higher for ILI case definitions than for SARI, ranging from 45.9% (95% CI: 44.4–47.4) for the ILI case definition in use before March 2012 to 48.3% (95% CI: 46.8–49.9) for the definition VW now uses. Although they were not included in this analysis, 18% (1,998/11,102) of VW specimens were first tested at local laboratories using less sensitive viral culture techniques. Since only positive samples are shipped to NICD for subtyping by rRT-PCR, it is likely that true positives are being missed, though the system reports case detections rates using samples tested at NICD only.

#### Representativeness

With public sentinel sites in just 4 out of 9 provinces, national representativeness was a relative weakness of the SARI programme. Though its six public sector sentinel sites were selected to represent a variety of geographic and demographic areas, the Cape provinces (i.e. Western Cape, Eastern Cape, and Northern Cape) are not represented. By comparison, representativeness was a relative strength of VW. Although VW sample collection diminished rapidly outside the regular influenza season, VW has excellent geographic and population coverage, with 205 sentinel sites servicing in all 9 provinces, including private sector general practitioners, primary care clinics, paediatric outpatient departments, and occupational health clinics.

#### Simplicity and Flexibility

Simplicity was relative weakness of the SARI programme; with an annual operating cost of approximately $500,000–800,000 USD, the system requires substantial investments in human resources and the training of dedicated personnel. Though the system collects extensive clinical and epidemiological data, it displays good flexibility, and has adapted to changing information needs by enhancing surveillance at selected sites to include patients with suspected or confirmed tuberculosis (TB) and patients with severe respiratory infection (respiratory symptoms >7 days).

Simplicity is a major strength of VW; participation of sentinel sites is voluntary, data and specimen collection materials are few, and minimal epidemiological data is collected. Laboratory tests account for the majority of the programme’s operating costs (approximately $100,000 USD per annum), while coordination and overhead costs are low (approximately $20,000 USD per annum) and can be scaled up or down as funds permit. VW also displays good flexibility, and has expanded from fewer than 20 sentinel sites in 2005 to 205 sites in 2012 with little growth in the programme’s overhead costs.

#### Acceptability and Stability

Indirect measures of acceptability include completeness and timeliness of data reporting; on each account, the SARI programme performs well. Specimens and forms are couriered to NICD to ensure transportation is timely and successful. Participation in the SARI programme is controlled through dedicated personnel and the high level of resources supporting the programme ensure stability. The willingness of VW sentinel sites to participate voluntarily suggests acceptability is also a relative strength of the programme, though many do not send samples regularly. In 2011, 21% (42/202) of sites sent fewer than 5 samples in the entire year. The number of participating sites also supports programme stability, though with few resources to support the system, VW specimens and forms are sent to NICD via private laboratories used by participating clinicians.

## Discussion

Surveillance for ILI and SARI is expanding rapidly in Africa; a review of 15 selected African countries between 2006–2010 showed substantial increases in the number of surveillance sites for both ILI (from 21 to 127) and SARI (from 2 to 98)[16]. For much of the past three decades, South Africa’s influenza surveillance activities have focused primarily on virological monitoring of mild influenza-associated disease. VW’s outpatient ILI surveillance was established to serve this purpose, aiming to guide vaccine strain selection and monitor the type, seasonality, and geographic distribution of influenza and other respiratory viruses. The system’s core strengths, especially national representativeness, support these objectives, though its reliance on voluntary practitioners limits the quality of data collected and VW is actively working to limit its sites to clinics that are performing well as part of the network. The recent establishment of SARI surveillance was intended to address the need for improved epidemiologic and virological monitoring of severe influenza-associated disease. Though the SARI programme is not nationally representative, the high quality and detail of data collected sheds light on the local burden and epidemiology of SARI, which is especially relevant from a clinical and public health standpoint and may be better suited to address questions of public health priority in South Africa.

With 30-years of historical seasonal influenza data, VW has helped to characterise seasonal influenza trends in South Africa[7]. Importantly, its lower operating costs are sustainable in an environment of competing health priorities and limited resources. This study has shown VW also detects the influenza season sooner than SARI, an advantage that can help to better inform the timing of national prevention and treatment policies, such as vaccination periods and the use of pharmaceutical and non-pharmaceutical interventions to control spread. With higher influenza detection rates than SARI, VW is also more efficient from the standpoint of laboratory testing, though it tests for fewer pathogens. Data quality was a relative weakness however. Without dedicated personnel at sentinel sites, patient interview forms were frequently incomplete and adherence to the ILI case definition in use since March 2012 was poor when it was first introduced. VW specimen collection also falls rapidly outside the regular influenza season. Without systematic data collection, the number of specimens collected may reflect numbers of participating sentinel sites rather than the intensity of influenza transmission, limiting its ability to establish reliable baseline levels of activity. This makes it difficult to accurately identify the end of the influenza season. Also, like SARI the VW programme does not estimate an epidemic threshold, though both are considering doing so and this would be a useful addition in the future.

The SARI system’s primary strength, data quality and depth, supports the programme in meeting its many surveillance objectives, which depend heavily on detailed epidemiological patient data. Understanding the local burden of SARI and the underlying risk conditions associated with severe disease and the use of health care resources is particularly important for clinical and public health decision making. For example, this information can be used to improve prevention and clinical management in high risk patients by prioritizing patients for vaccination and treatment[9]. It also allows for the study of the interaction between influenza and other priority diseases such as pneumonia, HIV[9] and TB[17]. With stable, year-round specimen collection, the system also provides data needed to establish historical trends, set epidemic thresholds, and understand the relation between virus strain and disease severity. This information can be used to rapidly assess the severity of each influenza season and unexpected events, as the recent A(H1N1)pdm09 pandemic demonstrated. The number of SARI specimens collected also reflects the burden of pneumonia, though this has many viral and bacterial aetiologies, each with different seasonalities. The system may also be used as a research platform for economic evaluations, though economic data is not routinely gathered.

Despite its many strengths, the SARI system has a few notable weaknesses; during the course of this evaluation, sentinel sites were located in just 4 of 9 provinces (excluding all of the Cape provinces), limiting its national representativeness. With its high operating costs and a history of dependence on external financial support, the long-term financial sustainability of the system has been uncertain. Encouragingly, the South Africa government has recently started supporting SARI surveillance. By acting as a platform for the surveillance of other respiratory illnesses, including tuberculosis and respiratory syncytial virus (RSV) infection, the SARI system integrates influenza surveillance with a broader approach to respiratory disease surveillance. This has several benefits: it allows for efficiencies in data collection and laboratory transport, and is a more efficient use of resources. These widespread benefits enhance the usefulness of the system as it meets its own surveillance objectives and broader national priorities. To improve national representativeness, expansion of the system may be required, although fewer sustainable sentinel sites should be preferred over a geographically widespread, but under-resourced, national network.

The choice of clinical case definition for influenza surveillance will depend on programme objectives; systems such as VW that aim to track seasonal influenza activity and collect virus isolates with limited resources may wish to maximize specificity, while systems such as SARI that are designed to estimate disease burden may wish to identify all influenza cases in a specific population and therefore prefer a more sensitive case definition. Previous studies have identified wide ranging sensitivity and specificity estimates for ILI [18–22], though varying populations and study designs limit direct comparability. VW’s current ILI case definition was found to be more specific than the definition previously used by the programme, which should help to reduce the number of specimens needed for each positive influenza case identified. The SARI case definitions for children age 2 days to <3 months and 3–59 months were more sensitive and less specific than estimates recently reported in children aged 2–59 months in Kenya[22].

The balance between surveillance for mild and severe influenza-associated disease should be determined by the information needs and priorities of each country. In settings with limited laboratory capacity, it may be desirable to test for influenza using a subset of ILI and SARI patients while gathering epidemiological data from a larger number of sites. With both the VW and SARI systems active, it is possible to study the contribution of influenza to both outpatient and inpatient respiratory disease. To meet long standing virological surveillance objectives—namely to determine the timing of the annual influenza seasons, conduct resistance testing and inform vaccine strain selection—it is not necessary to collect extensive epidemiological information or employ systematic sampling methods. However, the epidemiologic and virological monitoring of severe respiratory disease provides national benefits that are worth the added investment. In line with WHO recommendations[12], we propose that both outpatient ILI and hospitalized SARI should be under surveillance in South Africa to best monitor influenza activity. ILI surveillance should be limited to sites that are currently preforming well as part of the network and improved to be more systematically gathered, and SARI surveillance should be expanded to be more nationally representative, even if this requires scaling back on information gathered and numbers of specimens tested.

## Supporting Information

S1 Table Minimum data collection standards [12] for ILI and SARI surveillance. (DOCX)

S2 Table Influenza cases detected by subtype from SARI and ILI surveillance programmes (2009–2012), South Africa. (DOCX)

S3 Table SARI and ILI case definitions used for screening and enrolment by the SARI and Viral Watch surveillance programmes respectively, South Africa, 2009–2012. (DOCX)
