# Supplementary material for: Evaluation of Two Influenza Surveillance Systems in South Africa
Source: PLoS One. 2015 Mar 30;10(3):e0120226. doi: 10.1371/journal.pone.0120226 (PMC4379032; doi:10.1371/journal.pone.0120226)
Supplement: S1 Table — (DOCX) [file pone.0120226.s001.docx]

Table S1: Minimum data collection standards [12] for ILI and SARI surveillance.

| **Essential minimum data for ILI/SARI surveillance** |
| --- |
| **General information:** |
| Unique patient identifier used to track patient and link laboratory data to epidemiological data |
| Gender |
| Age |
| Date of symptom onset |
| Date of hospitalization (SARI patients only) |
| Date of specimen collection |
| Antiviral use for present illness at the time of specimen collection |
| **Clinical signs and symptoms:** |
| History of fever and body temperature at presentation |
| Pregnancy status |
| **Chronic pre-existing medical illness(es):** |
| Chronic respiratory disease |
| Asthma |
| Diabetes |
| Chronic cardiac disease |
| Chronic neurological or neuromuscular disease |
| Haematological disorders |
| Immunodeficiency, including Human Immunodeficiency Virus (HIV) or Acquired Immune Deficiency Syndrome (AIDS) |
| **Optional data (depending on programme needs):** |
| Signs and symptoms |
| Smoking history |
| HIV or AIDS recorded separately from other immunodeficiency |
| Tuberculosis co-infection |
| Body mass index (requires height and weight) |
| Influenza vaccination status (current and previous year) and date of vaccination |
| Ethnicity or member of disadvantaged minority |
| Specific haematological disorders (e.g. sickle cell disease) |
| Patient outcome |
