# Supplementary material for: Evaluation of Two Influenza Surveillance Systems in South Africa
Source: PLoS One. 2015 Mar 30;10(3):e0120226. doi: 10.1371/journal.pone.0120226 (PMC4379032; doi:10.1371/journal.pone.0120226)
Supplement: S2 Table — (DOCX) [file pone.0120226.s002.docx]

Table S2: Influenza cases detected by subtype from SARI and ILI surveillance programmes (2009-2012), South Africa

| **Surveillance system** | **Year** | **Influenza cases detected by subtype (%)** | | | | | |
| --- | --- | --- | --- | --- | --- | --- | --- |
|  |  | **A Unsubtyped** | **A(H1N1)** | **A(H1N1)pdm09** | **A(H3N2)** | **B** | **Total** |
| **Viral Watch (ILI)** | 2009 | 59 (3.4) | 4 (0.2) | 712 (40.5) | 863 (49.0) | 122 (6.9) | 1,760 (100) |
|  | 2010 | 1 (0.1) | 0 (0.0) | 211 (23.0) | 238 (25.9) | 468 (51.0) | 918 (100) |
|  | 2011 | 14 (1.2) | 0 (0.0) | 887 (74.6) | 160 (13.5) | 128 (10.8) | 1,189 (100) |
|  | 2012 | 10 (1.3) | 0 (0.0) | 7 (0.9) | 439 (56.7) | 318 (41.1) | 774 (100) |
| **SARI** | 2009 | 14 (3.5) | 0 (0.0) | 160 (40.5) | 194 (49.1) | 27 (6.8) | 395 (100) |
|  | 2010 | 0 (0.0) | 0 (0.0) | 45 (13.5) | 82 (24.6) | 207 (62.0) | 334 (100) |
|  | 2011 | 0 (0.0) | 0 (0.0) | 174 (38.9) | 113 (25.3) | 160 (35.8) | 447 (100) |
|  | 2012 | 5 (1.9) | 0 (0.0) | 1 (0.4) | 117 (45.5) | 134 (52.1) | 257 (100) |
