# Supplementary material for: Evaluation of Two Influenza Surveillance Systems in South Africa
Source: PLoS One. 2015 Mar 30;10(3):e0120226. doi: 10.1371/journal.pone.0120226 (PMC4379032; doi:10.1371/journal.pone.0120226)
Supplement: S3 Table — (DOCX) [file pone.0120226.s003.docx]

Table S3. SARI and ILI case definitions used for screening and enrolment by the SARI and Viral Watch surveillance programmes respectively, South Africa, 2009–2012

|  | **Date of use** | **Condition** | **Definition** |
| --- | --- | --- | --- |
| **SARI Surveillance Programme** | **February 2009 to present** | SARI in persons 2 days to < 3 months old | (i) Any child with diagnosis of suspected sepsis or physician diagnosed LRTI irrespective of signs and symptoms, AND (ii) patient presenting within 7 days of the onset of illness |
|  |  | Physician-diagnosed lower respiratory tract infection (LRTI) in persons 3-59 months of age | (i) Any child aged 3-59 months with LRTI, which includes bronchiolitis, pneumonia, bronchitis or pleural effusion, AND (ii) patient presenting within 7 days of the onset of illness |
|  | **February 2009 to April 2012** | SARI (≥5 years of age) | (i) Any person presenting with manifestations of acute lower respiratory infection, AND (ii) sudden onset of fever (>38ºC) AND (iii) cough or sore throat AND (iv) shortness of breath, or difficulty breathing with or without clinical or radiographic findings of pneumonia), or tachypnea, AND (v) patient presenting within 7 days of the onset of illness |
|  | **May 2012 to present** | SARI (≥5 years of age) | (i) An acute respiratory infection with history of fever or measured fever of ≥38°C, AND (ii) cough, AND (iii) onset within the last seven days, AND (iv) requires hospitalization |
| **Viral Watch Programme** | **March 2012 to present** | Influenza-like illness (ILI ) | (i) Patients of all age groups, with an acute respiratory illness with a measured temperature of ≥ 38 ˚C, AND (ii) cough, AND (iii) onset within the past 7 days |
|  | **2009 to February 2012** | Influenza-like illness (ILI ) | (i) Patients of all age groups, with acute respiratory tract infections of recent onset (within 72 hours), AND (ii) sudden onset of fever, AND (iii) two or more of headache, myalgia, cough and sore throat |
